# Intracystic papillary carcinoma of the breast

**DOI:** 10.2349/biij.1.1.e5

**Published:** 2005-07-01

**Authors:** M Muttarak, A Somwangprasert, B Chaiwun

**Affiliations:** Departments of Radiology, Surgery, Pathology, Chiang Mai University, Thailand

## HISTORY

A 44-year-old-woman presented with a painless lump in the right breast for 10 months. She had no nipple discharge and no familial history of breast carcinoma. She was treated at a provincial hospital by aspiration of the mass which revealed bloody fluid. She was informed that there was no abnormality. A few weeks later, she felt a lump again in her right breast, so she came to our hospital. Physical examination revealed a 3.5 cm, mobile mass in the upper outer quadrant of the right breast. There was no evidence of axillary lymphadenopathy. The patient was admitted for excisional biopsy of the breast mass.

## IMAGING FINDINGS

**Figure 1:**
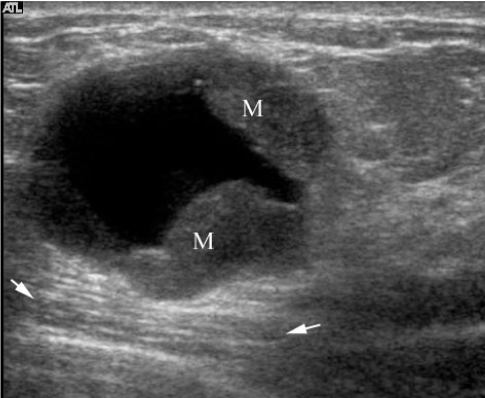
(a) Craniocaudal and (b) mediolateral mammograms show a 3.5 cm dense, round, partially circumscribed mass without calcification in the right upper outer quadrant.

**Figure 2:**
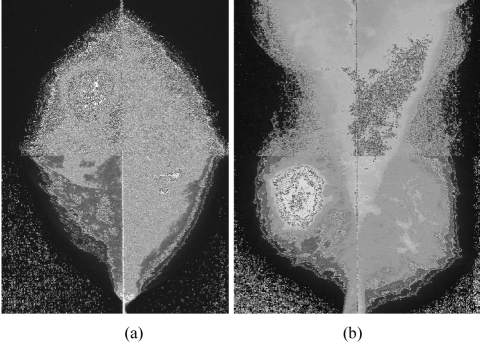
Transverse US image of the mass shows a complex mass with cystic and nodular solid (M) components. Posterior acoustic enhancement (arrows) is also seen.

**Figure 3:**
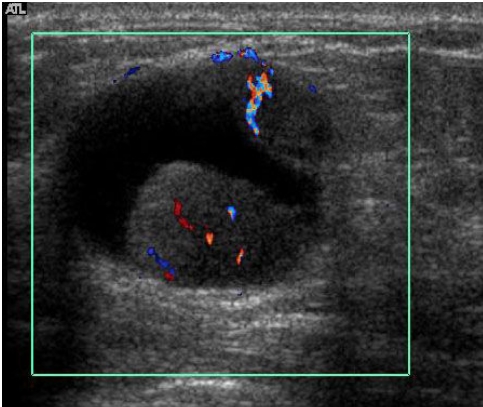
Transverse color Doppler US image shows vascular flow within the solid components.

Mammography showed scattered fibroglandular breasts with a 3.5 cm partially circumscribed, dense mass in the right upper outer quadrant (Fig 1). The mass contained no calcification. The axillary lymph nodes appeared normal. Ultrasonography (US) of the mass showed a complex mass with cystic and solid components (Fig 2). The mass also showed some posterior acoustic enhancement. Colour Doppler US demonstrated vascular flow within the solid component of the mass (Fig 3).

## PATHOLOGICAL FINDINGS

**Figure 4:**
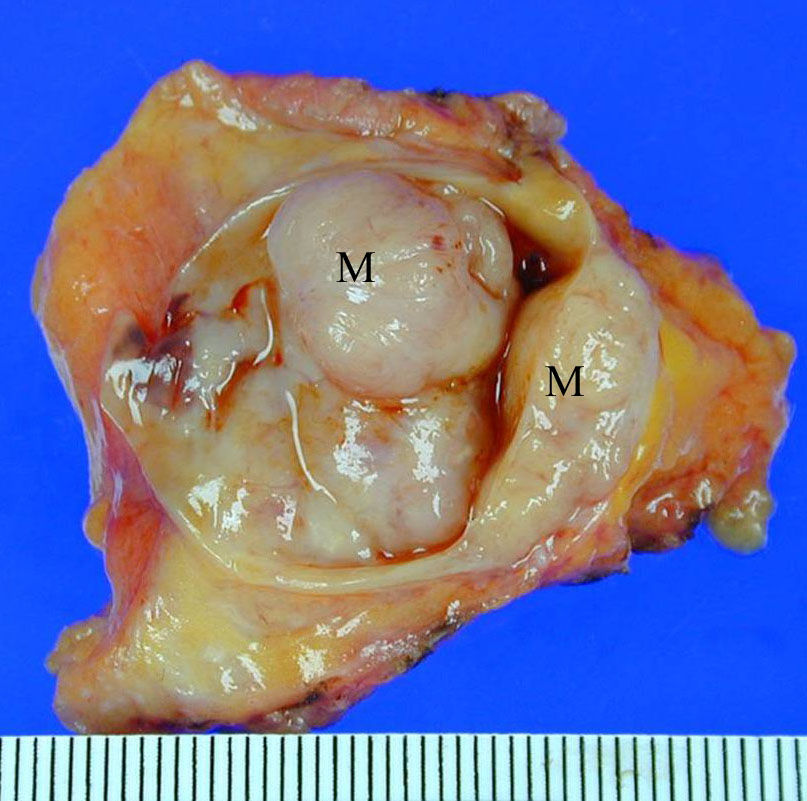
Photograph of the gross specimen shows a thick wall cyst with nodular lesions (M) protruding into the cystic space.

**Figure 5:**
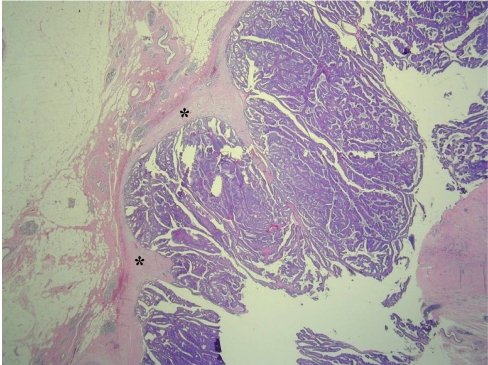
Low-power photomicrograph of a histopathologic specimen shows thick fibrotic cyst wall (*) and papillary fronds (Hematoxylin-eosin stain, original magnification, ×20).

**Figure 6:**
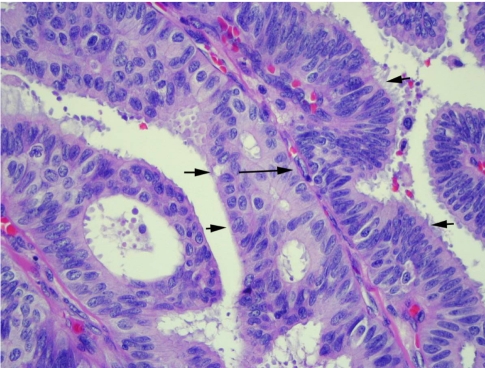
High-power photomicrograph shows a papillary frond (short arrows) with a fibrovascular core (long arrow). The epithelial cells are pleomorphic (Haematoxylin-eosin stain, original magnification, ×200).

**Figure 7:**
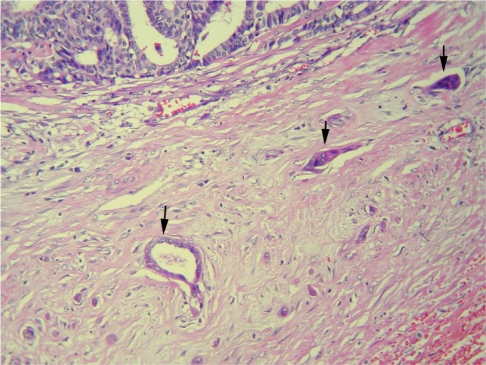
High-power photomicrograph shows invasive carcinoma (arrows) extending into surrounding fibrous stroma (Haematoxylin-eosin stain, original magnification, ×200).

At gross examination, the specimen contained a circumscribed mass measuring 3.5 cm with bloody content and solid nodular masses protruding into cystic space (Fig 4). At microscopy, there were multiple papillary fronds with thin fibrovascular cores. The columnar epithelial cells revealed a pleomorphic appearance (Fig 5-6). Invasive malignant cells were seen in the surrounding fibrous stroma (Fig 7).

## DISCUSSION

Papillary carcinoma of the breast is a rare malignant tumour, constituting 1-2% of all breast carcinomas in women [1]. Pathologically, papillary carcinoma has a frond-forming growth pattern supported by a fibrovascular stalk [1]. The absence of a myoepithelial layer distinguishes carcinoma from benign papillary lesion.The tumour may be solitary or multiple. The tumour is classified as papillary ductal carcinoma in- situ if the epithelium of a papillary carcinoma has features diagnostic of intraductal component. If a cystic component is present, the tumour is described as an intracystic papillary carcinoma [2]. Invasive papillary carcinoma occurs infrequently, often as only a small focus of stromal invasion and is almost always detected at the periphery of the lesion [2,3]. Papillary carcinoma generally occurs in older women aged 63-67 years [2-4]. Patients with papillary carcinoma may present with a palpable mass or bloody nipple discharge. The tumour may also be asymptomatic and identified at screening mammography. The tumour has a slow growth and a better prognosis than other forms of ductal carcinomas [1-8]. Axillary nodal metastases are infrequent.

On mammography, papillary carcinoma is seen as a round, oval or lobulated mass. The margins of the mass are usually circumscribed but may be obscured or indistinct.

On US, the tumour may appear as solid hypoechoic mass, or a complex mass with cystic and nodular solid components with posterior acoustic enhancement [2-4]. Haemorrhage within the cyst is frequently present due to ruptured capillaries within the cyst wall or haemorrhagic infarction of the tumor cells. Colour Doppler US is helpful to demonstrate the intramural blood flow, as in the presented case. Mammographically, differentiation between invasive and papillary ductal carcinoma in-situ is difficult. Papillary ductal carcinoma in-situ may manifest as single or multiple clusters of calcifications, sometime with dilated ducts, or single or multiple circumscribed masses. These findings are indistinguishable from those described in invasive papillary carcinoma [2,3,8-10]. Differentiation between in-situ and invasive papillary carcinoma is also difficult by fine needle aspiration or core biopsy because the centre of the lesion is often targeted, and invasion is often found at the periphery of the tumour. Therefore, excisional biopsy is often performed when papillary carcinoma is suggested.

Because papillary carcinoma has an excellent prognosis, the tumour may be managed by mastectomy or segmental resection. Axillary lymph node dissection or sentinal node biopsy is often performed in patients in whom invasion is likely [4,5,7,8]. Our presented case underwent mastectomy with axillary node dissection which revealed no residual tumour and no nodal metastasis in all 15 dissected nodes.
